# Metabolic Effects of Ketogenic Diets: Exploring Whole-Body Metabolism in Connection with Adipose Tissue and Other Metabolic Organs

**DOI:** 10.3390/ijms25137076

**Published:** 2024-06-27

**Authors:** Yusra Ahmad, Dong Soo Seo, Younghoon Jang

**Affiliations:** Department of Biology and Chemistry, Changwon National University, Changwon 51140, Republic of Korea; yusrahmad4304@gmail.com (Y.A.); mizar1105@naver.com (D.S.S.)

**Keywords:** ketogenic diet, adipose tissue, adipose tissue hormone, metabolic signaling, metabolic organ, cancer cachexia

## Abstract

The ketogenic diet (KD) is characterized by minimal carbohydrate, moderate protein, and high fat intake, leading to ketosis. It is recognized for its efficiency in weight loss, metabolic health improvement, and various therapeutic interventions. The KD enhances glucose and lipid metabolism, reducing triglycerides and total cholesterol while increasing high-density lipoprotein levels and alleviating dyslipidemia. It significantly influences adipose tissue hormones, key contributors to systemic metabolism. Brown adipose tissue, essential for thermogenesis and lipid combustion, encounters modified UCP1 levels due to dietary factors, including the KD. UCP1 generates heat by uncoupling electron transport during ATP synthesis. Browning of the white adipose tissue elevates UCP1 levels in both white and brown adipose tissues, a phenomenon encouraged by the KD. Ketone oxidation depletes intermediates in the Krebs cycle, requiring anaplerotic substances, including glucose, glycogen, or amino acids, for metabolic efficiency. Methylation is essential in adipogenesis and the body’s dietary responses, with DNA methylation of several genes linked to weight loss and ketosis. The KD stimulates FGF21, influencing metabolic stability via the UCP1 pathways. The KD induces a reduction in muscle mass, potentially involving anti-lipolytic effects and attenuating proteolysis in skeletal muscles. Additionally, the KD contributes to neuroprotection, possesses anti-inflammatory properties, and alters epigenetics. This review encapsulates the metabolic effects and signaling induced by the KD in adipose tissue and major metabolic organs.

## 1. Introduction

The term “ketogenic diet” (KD) typically describes a diet characterized by a minimal intake of carbohydrates, modest protein consumption, and a high fat intake [1]. The KD modifies protein utilization, as many proteins are taken up by the body for the process of gluconeogenesis while the remainder contribute to tissue repair [2]. This diet significantly reduces carbohydrate consumption, increasing fats and protein intake, which induces ketosis, a metabolic state in which the body relies on fats as its main energy source instead of carbohydrates. Reducing body fat and enhancing metabolic health are the main aims of following a KD [3]. Several studies have shown a decrease in body weight alongside an increase in whole-body energy expenditure of up to 10–15% in KD-fed mice [4]. The KD resulted in weight loss and a reduction in body fat mass compared to those on a standard control diet. Interestingly, the KD led to mild ketosis, characterized by an average circulating beta-hydroxybutyrate (BHB) concentration of approximately 2 mmol/L [5]. In both the KD and control groups, a low blood glycemic level was observed, whereas the KD group returned to normal glycemic conditions. By contrast, a decrease in body weight was observed in KD-fed rats due to the induction of ketosis [6]. Analysis of the percentage of body weight loss indicated that more than 10% of the initial body weight was lost by almost 96% of patients in the VLCK group within 2 months, compared with 3.8% of the patients in the LC-diet group. Additionally, the VLCK diet had a positive impact on body weight compared to the LC diet. In one study, after a 1-year follow-up, the VLCK diet was more effective in reducing body weight than the standard LC diet, with good adherence and mild, temporary side effects. Visceral obesity was specifically targeted by the KD [7]. Several studies have shown that the KD has a substantial effect on body weight and energy expenditure as a result of ketosis [8,9]. Together, these results reveal the efficiency of the KD in promoting weight loss in both rodents and humans and whole-body energy expenditure. KDs in human and animal studies are generally different. Human KDs include various categories, such as the classic KD, medium-chain triglyceride KD, modified Atkins KD, and low glycemic index KD [10]. By contrast, to ensure reliable laboratory results, the rodent models are subject to a more stringent KD. The compositions of commonly used diets, including KDs, are summarized in Table 1 [10,11,12,13,14,15,16,17,18].

**Figure 1:**
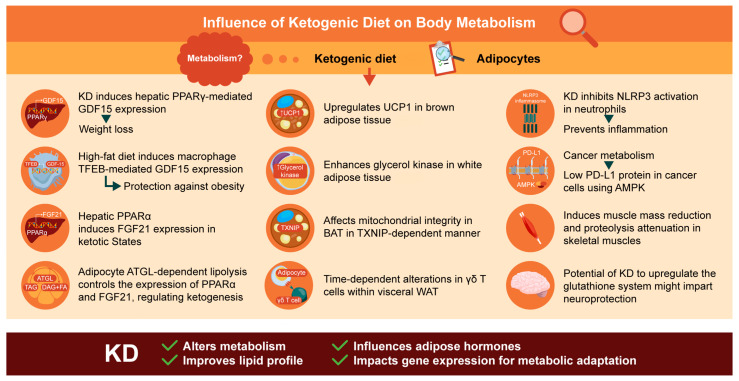
Highlights of the molecular aspects of each organ, including adipose tissue, on the impact of the ketogenic diet on body metabolism (red arrow indicates the effect of KD on molecular aspects; dark arrows indicate metabolic effects or molecular aspects).

In addition to its effects on weight loss, the KD reduces hyperlipidemia and certain cardiovascular risk factors [19]. One study demonstrated the effectiveness of the KD, which is high in fat and polyunsaturated fatty acids, in reducing the risk of being overweight and other chronic complications associated with obesity [20]. The applications of the KD are diverse and continually expanding. Its most established uses include obesity and refractory epilepsy; however, it can also be used as a treatment for neurological disorders, cancer, non-alcoholic fatty liver disease, type-two diabetes, and chronic pain, among others [21]. Furthermore, the KD has been found to augment cancer therapeutic responses and is considered safe as an adjuvant therapy alongside conventional radiation and chemotherapy [22]. Garbow et al. found that, in C57BL/6J mice, the expression of inflammatory cytokines and chemokines, along with the production of reactive oxygen species, was reduced, and in some cases suppressed, by the VLCK diet [23]. Some clinical data indicate that the KD positively affects circulating levels of interleukin (IL)-6 and -8, metalloproteinase-2, C-reactive protein, tumor necrosis factor-alpha (TNF-α), resistin, and lipid metabolism [24]. Numerous clinical studies have explored the effects of the KD on the incidence of obesity, highlighting the significance of experimental animal research in this field. In this review, we examined the effects of the KD on adipose tissue and other organs with highlights of the molecular aspects, as well as the metabolic alterations induced by the KD, as summarized in Figure 1. We began with an overview of whole-body glucose and lipid metabolism under the influence of the KD. We then focused on metabolic signaling and hormone mediators in adipose tissues and other key metabolic organs. Finally, we briefly discuss the role of the KD in regulating cancer cachexia and its impact on adipose tissue.

## 2. Glucose and Lipid Metabolism under the KD

The KD is known for its wide range of functions and applications, ranging from facilitating weight loss to improving various metabolic markers [25]. Various studies have shown a positive effect of the KD on insulin resistance in overweight or obese individuals [19]. Substantial weight loss related to the VLCK diet may play an important role in augmenting glycemic control [26]. In a study, patients with type-two diabetes who consumed a KD for 56 weeks demonstrated a significant reduction in body weight, body mass index, total cholesterol, low-density lipoprotein cholesterol, triglycerides, and blood glucose levels, as well as a significant increase in high-density lipoprotein (HDL) cholesterol [15]. Many studies have demonstrated that the KD markedly enhances glucose and lipid metabolism, thereby improving overall metabolic health [27]. Furthermore, the KD has proven to be versatile for numerous therapeutic interventions. Recent research has highlighted several benefits of the KD and LC diets, including improved glycemic control, decreased insulin resistance, and reduced inflammation. In addition to the KD, LC diets significantly reduce endogenous insulin requirements and lead to remarkably decreased postprandial glucose excursions [28]. Owing to reduced endogenous glucose production, fasting glucose levels also decrease, primarily resulting in low rates of glycogenolysis linked to an incomplete compensatory rise in gluconeogenesis [29]. The effects of LC diets on insulin sensitivity can vary depending on the patient, along with the overall state of energy balance and duration of treatment. In one of the studies, LC diets lessen insulin sensitivity, insulin secretion, and glucose tolerance [30]. Some studies have observed a substantial enhancement in insulin sensitivity with a LC diet, even in the absence of obesity [31,32]. The KD has become popular for the treatment of obesity, type-two diabetes, and non-alcoholic fatty liver disease [33]. By reducing the glycemic response induced by carbohydrates, the KD improved type-two diabetes mellitus and potentially reduced insulin resistance [34]. Furthermore, as inflammation plays a vital role in the pathophysiology of insulin resistance and type-two diabetes caused by obesity, the anti-inflammatory effect of the KD is directly related to reducing hepatic steatosis and enhancing overall body glycemic control [35]. Postprandial glucose levels are modified by carbohydrates and contribute to glycemic alterations and elevated insulin levels. Consequently, lowering carbohydrate intake leads to improved glycemic control, decreased insulin requirements, and insulin resistance [36]. Overall, changes in fat and protein content induced by the KD or LC diets result in a prolonged postprandial glycemic response and alterations in lipid metabolism, which influence insulin resistance and obesity.

Although the KD facilitates weight loss and improves various metabolic parameters, it has several side effects that may counterbalance its beneficial cardiovascular effects, owing to alterations in lipid metabolism [37]. KD intake has not only been shown to enhance glucose metabolism but numerous studies have also reported that the KD has a positive impact on lipid metabolism. A study observed that the KD decreased TG and total cholesterol levels, while increasing HDL levels, thus alleviating dyslipidemia. In one study, significant improvements were observed in both glucose and lipid metabolism following KD consumption, with the recruited participants exhibiting closely related characteristics [34]. The KD induces disruption of glucose and lipid metabolism, which may contribute to modifications in gut microbiota and metabolites [38]. In the study, both forms of the KD induced modification of the gut microbiota and associated metabolites. For instance, one study found that the impairment of lipid metabolism induced by two types of KD was linked to an increase in the occurrence of numerous amplicon sequence variants associated with *Ruminococcaceae* and *Lachnospiraceae*. In obese individuals, positive changes have been observed in TG and HDL levels induced by low-carbohydrate high-fat diets [39]. The KD prioritizes very low carbohydrate intake. This restriction affects sugar metabolism by regulating the rate of liver glycogen degradation, resulting in reduced blood glucose levels [40]. KD-derived ketone bodies (KBs), which are substitutes for metabolic substrates, elevate the intracellular concentration of glucose. Furthermore, it increases the mitochondrial acetyl-CoA concentration by circumventing the pyruvate dehydrogenase complex and supplying acetyl-CoA directly from acetoacetyl-CoA [41]. KBs play a fundamental role in the original Randle/glucose–fatty acid cycle. Intracellular glucose metabolism and oxidation are restricted by fatty acids and KBs and initiate insulin resistance and the prediabetic state [42]. Therefore, the impact of the KD on systemic metabolism remains unclear, necessitating further long-term studies using relevant animal models and clinical trials.

## 3. Insights and Mechanisms of Brown Adipose Tissue (BAT) in the Context of the KD

BAT is specifically designed for a process called thermogenesis, which involves heat production and requires an energy source [43]. For BAT, the principal fuel source for thermogenesis is the lipid obtained from its sources, stored in white adipose tissue, or from the diet. Therefore, BAT thermogenesis is a constituent of the overall energy expenditure, which specifically implies the combustion of lipids [44]. The KD is related to enhancements in lipid homeostasis, including the upregulation of fatty acid oxidation and utilization, as well as a reduction in lipid synthesis [45]. In addition to their roles in energy storage and thermogenesis, WAT and BAT function as endocrine organs and express constituents of the renin-angiotensin system [46]. The whitening of BAT can notably worsen by acetylation of PPAR*γ* in adipocytes, exacerbating age-related metabolic dysfunction [47]. The KD affects mitochondrial integrity in BAT through a TXNIP-dependent pathway [48]. UCP1, which is found in various cells in WAT, generates heat by uncoupling electron transport from ATP synthesis through UCP1 in BAT. It has been postulated that the increase in UCP1 levels is an outcome of the activation of the PPAR system of transcription factors [49]. Furthermore, SIRT1 stimulated the inhibition of mTOR, resulting in increased gene expression linked with autophagy and lysosomes in brown adipocytes, leading to resulting mitochondrial turnover and biogenesis and improved thermogenesis in BAT [50]. The KD in the BAT of the mice enhanced indicators of sympathetic activity (cAMP and CREB levels). The KD did not lead to a decrease in the lipid content and the weight of the BAT, whereas ketone ester significantly reduced both. This divergence might be due to the higher lipid content and increased calorie intake on the KD [51]. Activation of BAT results in the elevation of the *UCP1* gene through the influence of several transcriptional factors, including the PPAR and CCAAT/enhancer-binding protein families, cAMP response element-binding protein, and thyroid hormone [52]. Fatty acids can directly activate UCP1 activity and can also act as a fuel for thermogenesis [53]. Mechanisms dependent on UCP1 play a main role in enhancing obesity, glucose homeostasis, and BAT activity [54]. Contrary to WAT, BAT employs fats for the process of thermogenesis. Interestingly, by oral administration of D-β-hydroxybutyrate-(*R*)-1,3 butanediol monoester (ketone ester) in diet-induced obese participants, elevated BAT UCP1 levels and enhancement in insulin resistance were observed [55]. Together, these findings emphasize that under the KD, the activation of UCP1 can modulate BAT activity, facilitated by factors, such as PPAR*γ* and ketone esters, highlighting its capacity to address metabolic dysfunction and obesity-related complications.

## 4. Metabolic Regulation of WAT by the KD

The KD elevated UCP1 content and *UCP1* gene expression in both the WAT and BAT of mice and rats [51,56]. This increase in WAT UCP1 is indicative of a process called browning, in which WAT acquires structural and functional characteristics similar to those of BAT. CD36 is well known for its effects on fatty acid uptake and metabolism and these effects require its movement to the plasma membrane, which can happen rapidly in response to insulin or AMPK [57]. Under normal conditions, WAT has low UCP1 expression, but when compelled to undergo browning, it shows elevated UCP1 expression and increased energy expenditure [58]. In obesity, de novo lipogenesis is downregulated in WAT and renewing de novo lipogenesis in WAT reverses obesity-induced insulin resistance [59]. In mice, significant changes in gene expression and dietary-induced obesity are caused by KD feeding. In KD-fed ob/ob mice, in the absence of weight loss, similar alterations have been found, involving a marked elevation in the expression of lipid oxidative stress and repression of lipid synthesis genes [60]. Surprisingly, as demonstrated by Zhang et al., a KD combined with exercise reduced PPARγ and lipid synthetic genes while, in participants on a KD without exercise, it improved the PPARα and lipid β-oxidation gene program in the liver [61]. Additionally, activation of the AMPK/SIRT1 axis in the energy metabolism sensing pathway increases the deacetylation of PPARγ, promoting the restructuring of adipose tissue and increasing the expression of UCP1 [62]. Glucose transporter 1 is the major glucose transporter in the blood–brain barrier. A lack of glucose transporter 1 leads to hypoglycorrhachia and impairment of cerebral energy metabolism, resulting in serious developmental delays, seizures, and complex motor disorders [63]. In the subcutaneous WAT, a decrease in adipocyte size improves insulin sensitivity in obese individuals. In visceral but not subcutaneous WAT, the connection between adipocyte hypertrophy and inflammation facilitated by M1-like macrophages and/or B-cells may be involved in insulin resistance [64]. Some studies have indicated that in WAT, renin-angiotensin system activation has a significant impact on insulin sensitivity and inflammation and plays an important role in the progression of metabolic disorders associated with obesity [46]. In conclusion, KD interventions exhibited multifaceted effects on adipose tissue metabolism, including the activation of WAT browning and increased lipolysis, modifying gene expression profiles. These findings underscore the complex interplay between dietary interventions and metabolic regulation and highlight the importance of further research in this field.

## 5. Gene Expression Profiles of Metabolic Enzymes under the KD

The KD has abundant positive effects on inflammation, metabolism, and hypertension, which may be associated with gene transcription modulation through histone β-hydroxybutyrylation, a histone post-translational modification facilitated by BHB [5]. Additionally, regardless of the high intake of saturated fat, serum lipids levels did not increase. The inhibition of transcription factors and enzymes involved in lipid synthesis is a remarkable aspect of gene expression, including fatty acid synthase, stearoyl-CoA desaturase-1, and sterol regulatory element-binding protein-1c [65]. Studies have indicated that methylation can affect both the development of obesity and individual responses to dietary weight loss strategies. It has been observed that adipogenesis-associated mechanisms are modulated by methylation [66]. Moreover, variations in methylation status have also been observed in some vital genes, including *ZNF331*, *FGFRL1*, *CBFA2T3*, *C3ORF38*, *JSRP1*, and *LRFN4*, whose methylation is particularly linked to weight loss and/or ketosis induced by VLCKD [67]. The KD decreases PD-L1 protein abundance in cancer cells in an AMPK-dependent manner [68]. However, across various tissues, a distinct methylation profile is linked to obesity and the methylome associated with obesity is interconnected with complexities, such as insulin resistance and cancer [69]. The complexities in the methylation profiles within various tissues highlight the interplay between obesity, insulin resistance, and cancer susceptibility, underscoring the necessity for further investigation of the prolonged impact of the KD on overall health.

KD-fed rats displayed high levels of reduced glutathione in mitochondria and an increased overall proportion of reduced-to-oxidized glutathione in the hippocampus. The potential of the KD to upregulate the glutathione system might impart protection against brain damage induced by seizure [70]. KBs may also affect microRNAs (miRNAs); in KD-treated participants, nutrient metabolism-linked genes, such as *mTOR*, PPARs, insulin, and cytokines, were distinctly targeted by miRNAs, demonstrating that the KD may alter the expression of miRNAs, resulting in a reduction in inflammatory ILs, such as IL-1β and IL-6, thereby diminishing neuroinflammation [71]. At the molecular level, KBs, such as BHB and acetoacetate, have been shown to affect epigenetic markers. This is achieved by inhibiting histone deacetylase 1, altering proteins via butyrylation at the post-translational level, affecting DNA methylation, and acetylating both histone and non-histone proteins [72]. Additionally, KBs regulate gene expression by managing metabolic processes and serving as signaling metabolites [73]. The initiation of histone hyperacetylation by BHB is related to a widespread modification in transcription, including the induction of oxidative stress resistance genes, such as forkhead box O3a, MT2, SOD2, and catalase. However, according to various studies, BHB altered the lysine β-hydroxybutyrylation of histones but had no significant impact on HDAC activity and histone acetylation [74]. The KD has a substantial influence on various molecular pathways associated with metabolic regulation. KBs, such as BHB, induce changes in histone modification, oxidative stress resistance genes, and transcriptional regulation; however, further research is necessary to elucidate the underlying mechanisms.

## 6. Metabolic Signaling by Fibroblast Growth Factor 21 (FGF21) and Growth Differentiation Factor 15 (GDF15) under the KD

Insulin, glucagon, and leptin are the primary hormones governing glucose and lipid metabolic regulation and are pivotal regulators of systemic metabolism. These hormones facilitate the majority of metabolic signaling, predominantly within metabolic tissues. Moreover, recent studies have highlighted the role of glucagon-like peptide 1, an incretin hormone produced in intestinal L cells, which influences a broad spectrum of metabolic tissues [75]. Semaglutide, a drug developed by Novo Nordisk, has significant therapeutic effects and is used in obesity treatment [76]. In the realm of adipose tissue, obesity, and hormones related to systemic metabolism, FGF21 and GDF15 have recently been recognized as important mediators of metabolic signaling between adipose tissue and other metabolic organs [77]. In this review, we extensively discuss these two factors and explore their interactions with the KD.

### 6.1. FGF21

FGF21, a member of the fibroblast growth factor family, is expressed in the liver, endocrine and exocrine pancreas, and adipose tissues [78]. KD-treated FGF21 knockout mice experienced weight gain and developed hepatic steatosis, whereas control mice showed weight loss and reduced fatty liver [79]. FGF21 plays a role in regulating lipid metabolism, indicating that a lack of FGF21 may lead to modified lipid metabolism and abnormal storage of liver fat in mice lacking FGF21 [80]. In brown adipocytes, FGF21 increased glucose uptake, oxidation, and thermogenic activation [81]. Furthermore, FGF21 is stimulated by the KD [82] FGF21 levels, in association with ATF4/5 TFs, depend on the nutrient context, including the KD [83]. In WT animals, FGF21 acts as a metabolic regulator, which plays a crucial role in facilitating the effects of the KD. Other studies have reported that PPARα is a primary regulator of hepatic FGF21 in mice [60]. Recently, Inagaki et al. observed hypotrophy and enhanced lipolysis in the adipocytes of FGF21 transgenic mice. In cultured adipocytes, lipolysis is activated by FGF21. These results indicate the potential function of FGF21 in WAT lipolysis [84]. Treatment with FGF21 enhances insulin sensitivity and reduces the expression of mammalian target of rapamycin complex 1 (mTORC1) within the same timeframe, demonstrating the potential interplay between FGF21 and mTORC1 to facilitate glucometabolic signaling [54]. FGF21 has emerged as a major mediator of the metabolic effects of the KD. FGF21 expands into brown adipocytes, enhances glucose uptake and oxidation, and initiates thermogenic activation. Studies have suggested that FGF21 participates in adipocyte hypotrophy and increases lipolysis, highlighting its potential importance in WAT metabolism. Overall, our findings emphasize the varied involvement of FGF21 in metabolic control and its potential therapeutic outcomes.

### 6.2. GDF15

GDF15 is a unique member of the transforming growth factor β superfamily and serves as a hormone, stress-induced cytokine, or stress-sensitive circulating factor [85]. Multiple reports and recent work have repeatedly shown a decreased intake of food in animals fed a KD, indicating a potential association between GDF15 and the management of obesity by the KD [86]. In mice, a decrease in oxidative phosphorylation in adipose tissue elevates the secretion of GDF15 [87]. In mice fed a high-fat diet, the expression of GDF15 mRNA was enhanced in both the liver and adipose tissue. Enhanced insulin sensitivity and protection against diet-induced obesity have been demonstrated in transgenic mice over-expressing GDF15 [88]. A recent study emphasized the significance of endogenous GDF15 induction in adipose tissue macrophages in obese mice. This induction represents an adaptive response to lysosomal stress associated with obesity to inhibit inflammation in adipose tissue and insulin resistance, irrespective of any modulation in food intake [77]. Additionally, in response to thermogenic stimuli, brown and beige cells released GDF15 as an adipokine, which reduces pro-inflammatory signaling by interacting with macrophages [89]. The transcription factor EB (TFEB)–GDF15 axis is essential in adapting to obesity-induced metabolic stress; GDF15 acts as a ‘lysokine’ released in reaction to lysosomal stress via TFEB activation. By improving lipid breakdown and decreasing adipose inflammation, TFEB-induced GDF15 protects against diet-induced obesity and insulin resistance [90]. Its association with TFEB activation further highlights its importance in metabolic adaptation. To encourage GDF15 release in HFD-fed mice, Day et al. used metformin and observed that the food intake of these mice was lower than that of the control group. This alteration was not observed in GDF15 knockout mice, suggesting that increased levels of GDF15 decrease food intake [91]. The diverse roles of GDF15 show its versatility in managing physiological responses, including lipid catabolism, inflammation inhibition, and insulin sensitivity. The complex interaction between GDF15, macrophages, and adipose tissue underscores the complexity of metabolic regulation and offers opportunities for further research to clarify its precise mechanisms and therapeutic uses in metabolic disorders.

## 7. The KD: Interconnections between Adipose Tissue and Other Organs

In addition to affecting adipose tissue, the KD influences a wide range of metabolic tissues, including the liver, muscles, brain, and immune system. Recent studies have indicated that various adipokines, functioning as adipose tissue hormones, play important roles in organ crosstalk and the regulation of systemic metabolism [92,93]. The KD is a dietary regimen that drastically restricts carbohydrate intake and increases fat consumption, thereby promoting systemic metabolic regulation that utilizes fat as a primary source of energy. In the context of the KD, it appears that metabolic organs interact with the adipose tissue to regulate whole-body metabolism. Given that the KD is currently under clinical investigation as an anticancer dietary intervention [22], its influence on adipose tissue and metabolic regulation in tumor tissues is considered highly significant. In this review, we discuss the metabolic effects of the KD and its metabolic changes on various organ-specific diseases, as summarized in Table 2.

### 7.1. Liver

The liver serves as a primary sensor that regulates nutritional status and conveys signals to other tissues, including adipose tissue, to maintain overall metabolic homeostasis. In a previous study, 6 days of an identical KD showed a 22% reduction in liver volume and 70% of this decrease was linked to a deficiency in glycogen. Furthermore, the decline in endogenous glucose production was due to hepatic glycogen depletion, as suggested by the unchanged levels of hepatic pyruvate carboxylase [33]. Significantly, weight reduction has been linked to a decrease in liver enzyme levels and improvements in histological factors related to liver steatosis, inflammation, and fibrosis [119]. However, it has been suggested that a very low carbohydrate intake can be responsible for a significant reduction in insulin secretion and possibly an accelerated reduction in visceral adipose tissue (VAT) and liver fat accumulation. In obese individuals undergoing weight loss interventions, longitudinal alterations in VAT and liver fat have been reported in quantitative imaging-based MRI scans [120]. Luukkonen et al. reported that during intrahepatic ketone production, high levels of liver TG hydrolysis were observed in patients on VLCK because of a decline in circulating insulin concentrations, endogenous glucose production, and hepatic insulin resistance [33]. VLCKs could serve as a secure and effective short-term strategy to achieve a prompt reduction in VAT and liver fat content [121]. The results indicated that the mice model of diabetes showed a relative increase in liver weight, whereas KD treatment reversed this effect. In the KD group, serum total cholesterol, TG, and HDL levels were significantly elevated [122]. This study illustrated that in the low-carbohydrate high-fat group, the weight of the liver was reduced in conjunction with an elevated ratio of fat mass to lean mass. Therefore, the endocrine and metabolic changes induced by the KD may further influence the development of hepatic steatosis through the modulation of various processes within the adipose-tissue-liver axis [95]. CD36 plays a pivotal role in lipid intake. KD-treated mice show a significantly high level of CD36. Similarly, the prominent expression of the CD36 protein on hepatocyte membranes was markedly increased in the KD group compared to the other groups [96]. In one of the recent studies, the KD dramatically increased the mRNA expression of CD36, the main receptor involved in hepatic lipid intake [123]. Therefore, the KD may offer a novel and practical mouse model for the study of fatty liver diseases. However, further research is required to investigate the effects of the KD on adipose tissue under thermoneutral conditions.

The regulation of liver metabolism by the KD plays a crucial role in controlling adipose tissue function and modulating various key metabolic signaling pathways and gene expression. Additionally, mTORC1 is the main modulator of cell metabolism and a strong inhibitor of autophagy [97]. Interestingly, in the livers of KD-fed mice, the signaling of mTORC1 was observed to be inhibited [124]. The KD has been shown to elevate the activity of transcription factors, such as forkhead box O3 and p53, which activate a range of genes linked to autophagy in the mouse livers. Moreover, following a KD for a minimum of 3 days can stimulate ketogenesis and expression of hepatic FGF21 [98]. The intensity of hepatic steatosis is closely linked to the flux of free fatty acids (FFAs) generated from the adipose tissue [125]. This relation evolves because FFAs transported to the liver undergo esterification into TG within the hepatocytes. The increased distribution of FFAs impairs liver insulin sensitivity, which prompts the transcription of sterol-responsive element-binding protein 1c [126]. Growing evidence indicates that CD36 not only functions as a FFA transporter but also influences FFA oxidation, lipid synthesis, inflammation, and autophagy in the liver cells [127]. These processes include lipolysis, de novo lipogenesis, lipid uptake, beta-oxidation, ketogenesis, and lipid excretion. A recent study has demonstrated that a KD leads to weight reduction and induces non-alcoholic steatohepatitis and liver fibrosis when administered under thermoneutral conditions [128]. This enables de novo hepatic lipogenesis, which aggravates hepatic steatosis. Leptin exhibits dual effects by decreasing lipid accumulation and lipotoxicity, thus exerting an anti-steatotic effect [129]. Despite the increase in hepatic triglyceride levels, no elevation in fibrosis or steatosis has been reported on liver histology. Interestingly, a recent study showed that KBs alleviated KD-induced hepatic steatosis through the expansion of eWAT [130]. Additionally, their observations revealed that the liver releases the KB, BHB, in response to metabolic stress. PPARγ has been shown to promote the expansion of eWAT in response to metabolic adaptations through its acetylation-dependent mechanism.

### 7.2. Cardiac and Skeletal Muscles

The KD improves cardiac function and mitigates cardiac remodeling in diabetic mice with severe diabetic cardiomyopathy [73]. CD36 plays a fundamental regulatory role in cellular lipid metabolism, particularly within the cardiac muscle [131]. According to a most remarkable observation, a 50% reduction in myocardial glucose uptake was due to the elevated level of KBs, reaching up to 3.8 mM, regardless of maximal insulin stimulation and sufficient supply of glucose. Therefore, even when both substrates were available, a preference was observed for KBs over glucose [99]. The oxidation of KBs is cataplerotic, which results in the depletion of intermediates in the Krebs cycle and the weakening of metabolic efficiency. To balance this cataplerotic effect, some anaplerotic substances, such as circulating glucose, glycogen, and glucogenic amino acids, are important and pyruvate carboxylase is an important anaplerotic enzyme in the heart [100]. Adiponectin, obtained from the adipose tissue, exhibits antioxidant properties in cardiomyocytes by inhibiting myocardial NADPH oxidase activity [101]. This inhibition occurs via the prevention of the AMPK-mediated membrane translocation of RAC1 and p47phox (NCF1) [132]. This may act as a protective mechanism against harmful arrhythmias and other adverse effects arising from oxidative stress in the myocardium [133]. The vital role of epicardial adipose tissue is to secrete adiponectin from epicardial adipocytes [102]. Additionally, it promotes fibrogenesis through its pro-inflammatory effects. However, in response to the scarcity of dietary glucose, some organisms attempt to maintain normal plasma glucose by increasing cardiac muscle ring-finger protein 1 expression and reducing the expression of cardiac GLUT4 [134]. Adiponectin shields coronary circulation, improves endothelial function, decreases oxidative stress, and indirectly reduces IL-6 and C-reactive protein.

Hans Krebs coined the term ‘physiological ketosis’ to distinguish the diet-induced metabolic state from pathological diabetic ketosis [135]. During periods of low glucose accessibility, the physiological purpose of ketosis is to maintain muscle and central nervous system functions by utilizing the high-energy metabolic substrate offered by KBs, allowing ketones to survive and remain efficient despite a lack of glucose [103]. According to Fournier et al., the significant restoration of glycogen levels can occur even in the absence of carbohydrate intake, despite substantial amounts of exercise, with lactate, glycerol, and amino acids as likely carbon sources [104]. The KD resulted in the reduction in muscle mass, integrated by decreased plasma insulin and IGF-1 levels, and elevated plasma corticosterone levels. Subsequently, there was an augmentation of ubiquitin ligases marked by glucocorticoid receptors and genes associated with autophagy in the Ga, TA, and Sol muscles. Hu et al. noted that elevated endogenous glucocorticoids and impaired insulin signaling are required for the stimulation of muscle proteolysis [105]. Increased levels of KBs lead to a reduction in the utilization of glucose by peripheral tissues, anti-lipolytic effects in adipose tissue, and the possible attenuation of proteolysis within skeletal muscles [106]. Skeletal muscle contraction facilitates the translocation of the FAT/CD36 protein from the intracellular site to the cell membrane. This highlights its function as a flexible regulator of intracellular fatty acid absorption during increased metabolic demands [136]. Hepatic lipid oxidation and lipolysis are elevated by leptin in the skeletal muscles and adipocytes [107]. Leptin, produced and secreted by the adipose tissue, can influence smooth muscle cells, potentially contributing to pro-atherosclerotic effects [137]. Moreover, elevated leptin levels rapidly restrain insulin-mediated glucose absorption in rat skeletal muscle cultures, with no impact on the translocation of GLUT4 [138]. Finally, and unexpectedly, in skeletal muscle, the rate of myofibrillar protein synthesis is reduced by the administration of leptin, particularly with regard to protein metabolism [108]. The role of leptin derived from adipose tissues in cardiovascular and muscle metabolism remains unclear [139], necessitating further research to elucidate the functions of novel hormones derived from adipose tissues in muscle metabolism.

### 7.3. Neurological Disorders

Several studies have illustrated the processes by which the KD may alter brain excitability and reduce the frequency of seizures [109]. Following KD consumption, the concentration of KBs increases in the blood and cerebrospinal fluid. Within the first week of initiating the KD, a gradual progression was observed as the levels of ketones in the serum consistently increased [110]. In the past, the KD was used as the primary treatment for epilepsy in pediatric patients. In different animal models, the progression of chronic seizures has also been observed owing to the effect of the KD [111]. In a recent study utilizing a mouse model of malignant glioma, the KD reduced tumor growth and improved survival, which were related to decreased levels of ROS in tissues [140]. These findings indicate that the KD interrupts adenosine A1 receptor signaling and diminishes seizures. This is evident from the reduction in electrographic seizure-like activity in transgenic mice overexpressing neuronal adenosine kinase or lacking the adenosine A1 receptor [112]. In a recent study, Hui et al. performed a statistical analysis of the labeled circulating metabolites and reported that the brain mainly utilizes glucose and lactate during the KD [141]. This conclusion conflicts with the common belief that KBs are a fundamental energy source for neuronal cells during the KD. Interestingly, resistin is a peptide hormone originating from the adipose tissue and is defined by its abundance in cysteine [142]. For the synthesis of pro-inflammatory cytokines, CD36 induces the heterodimeric binding of toll-like receptors (TLRs), and the subsequent activation of the Nf-kB transcription factor [143]. Resistin is known for its substantial role in the central nervous system, especially in controlling pituitary somatotroph cell functions, influencing hypothalamic and peripheral insulin responsiveness, thermogenesis, and feeding behavior and amplifying renal sympathetic nerve activity [113]. One study found that the KD may influence the levels of several pro-inflammatory adipokines, such as leptin, chemerin, and resistin, in children with refractory epilepsy [144]; however, the implications of these findings remain unclear.

### 7.4. Inflammation

The activation of NLRP3 inflammasome and IL-1β production is inhibited by lipopolysaccharide in macrophages, which protect against muscle loss [145]. This suggests a potential therapeutic target for combating pathophysiological conditions associated with inflammation. After following an isocaloric KD, elevated BHB and decreased IL-1β levels were observed. Additionally, high levels of FGF21 and lower IL-1β levels were identified in the serum [146]. Adipose tissues consist of immune cells that control their function in both health and disease. Under healthy conditions, immune mechanisms facilitate the maintenance of tissue homeostasis. In obesity, the immune profile of the adipose tissue is modified, leading to a chronic low-grade inflammatory state, which eventually becomes systemic and induces insulin resistance and metabolic disease [147]. Goldberg et al. demonstrate that short-term feeding of the KD alters the content of the innate immune cells in visceral adipose tissue, leading to reduced inflammation and enhanced biomarkers of metabolic health [148]. The KD inhibits NLRP3 activation in neutrophils to prevent inflammation [149]. A later study demonstrated that in adult animals, anti-inflammatory effects were stimulated by microglial ramification induced by BHB [150]. In another study, in rat models of spinal cord injury, administration of a KD before the injury relieved neuroinflammation by inhibiting microglial activation and converting microglia from the M1 to the M2a phenotype [42]. In KD-feeding mice, hepatic inflammatory markers, IL-1, TNF-α, and CCL-2, were elevated as compared with observations in the chow diet [5,151]. However, in the adipose tissue, IL-6 levels were significantly decreased while PAI-1 levels were noticeably higher. After 22 weeks of KD feeding, MCP-1, IL-1β, and IL-6 were identified to be increased in the blood. Recent studies have shown that BHB suppresses the NLRP3 inflammasome in macrophages and neutrophils, which is a significant feature of the KD in improving lifespan and health [152,153]. The KD exerts anti-inflammatory effects in diverse physiological contexts. Research demonstrates that the KD induces up-regulation of BHB levels associated with decreased levels of pro-inflammatory cytokines, such as IL-1β, and appears to inhibit the activation of the NLRP3 inflammasome. Suppression of inflammatory pathways indicates that the KD has potential efficacy as a valuable approach for addressing a range of pathophysiological conditions linked to inflammation.

### 7.5. Cancer Cachexia

Cancer cachexia is an extreme wasting syndrome that is distinguished by decreased food consumption and ultimate weight loss. It affects almost 80% of patients with cancer, resulting in considerable morbidity and mortality [154]. Cancer cachexia is typically characterized by imbalanced metabolism, catabolism, and skeletal muscle degeneration and involves nutritional control through diverse mechanisms [112]. Various clinical and experimental studies have revealed abnormal physiological processes, including extreme fatty acid oxidation and rapid deployment of lipids from the adipose tissue. Thoroughly, this reveals the evolving nature of adipose tissue at the time of the progression of cachexia, including processes linked to the tumor microenvironment. During lipogenesis, excess non-esterified fatty acids are converted into TGs and are eventually stored in the cytosolic lipid droplets of adipocytes [114]. In cancer cachexia, alterations in lipid metabolism lead to a substantial decrease in total fat mass, accompanied by increases in lipolysis, fatty acid oxidation, and hyperlipidemia [155]. Furthermore, cachexia is correlated with a reduction in the lipogenic rate and diminished performance and expression of lipoprotein lipases [115]. During cachexia, extensive wasting of WAT is repeatedly observed and evidence suggests that it introduces the breakdown of proteins in the skeletal muscle or any reduction in food intake.

In contrast, in both human and mouse models of cancer cachexia, a persistent state of metabolic stress results in elevated cytokine levels owing to an energy imbalance across multiple organs [116]. In the adipose tissue during cachexia, numerous pro-inflammatory factors, such as TNFα, IL-1β, and IL-6, are stimulated [156]. In both the initial and later stages of cachexia, a previous study found a positive connection between serum IL-6 and FFA, indicating that in cachexia, weight loss might be induced by IL-6 by accelerating WAT lipolysis [117]. In cancer cachexia, elevated lipolysis and fat oxidation, reduced lipogenesis, impaired lipid deposition and adipogenesis, and browning of WAT can cause adipose atrophy [114]. Research on cachexia in rodents proposed that the initiation of thermogenesis, including elevated mitochondrial uncoupling of oxidative phosphorylation and heightened fatty acid β-oxidation in BAT, could be a factor in increased whole-body lipid utilization in cachexia, at least in later stages of cachexia [118]. Collectively, cytokines derived from the adipose tissue are likely to play important roles in the development of cancer cachexia.

## 8. Conclusions

The KD demonstrates diverse applications, showcasing its efficacy in body weight reduction and various therapeutic interventions. The adaptability of the KD is demonstrated by its wide-ranging applications, which extend to efficiently reduce body weight and address diverse therapeutic requirements. The KD also helps improve both glucose and lipid metabolism, potentially easing dyslipidemia and obesity-related complications. According to various studies, the KD has the potential to manage hyperglycemia, insulin resistance, and obesity, with possible benefits for lipid profiles and glycemic control. The KD promotes glucose and lipid metabolism, decreases TGs and total cholesterol while elevating HDL levels, and alleviates dyslipidemia. The oxidation of ketones depletes intermediates in the Krebs cycle, requiring anaplerotic substances, such as glucose, glycogen, or amino acids, to maintain metabolic efficiency. However, the underlying mechanisms behind the systemic metabolic changes induced by the KD remain unclear.

KD-mediated changes in adipose tissue represent significant metabolic events. BAT plays a crucial role in thermogenesis and lipid combustion and BAT UCP1 levels are altered by dietary factors, including the KD. Furthermore, WAT browning and elevated UCP1 expression in both white and BATs have been promoted by the KD. The KD induces lipolysis in WAT, resulting in the release of non-esterified fatty acids into the bloodstream via diminished circulating insulin levels. The effect of the KD has been observed in various organs, including the liver, skeletal muscle, and heart, among others. The KD induces muscle mass reduction, potentially involving anti-lipolytic effects and proteolysis attenuation in skeletal muscles. However, within skeletal muscle, due to the elevated levels of KBs, a reduction in protein breakdown occurs. The ultimate contributions of the KD have been observed to be neuroprotection, anti-inflammatory effects, and epigenetic modifications. The KD significantly increased the concentration of ketones in the blood and cerebrospinal fluid, potentially reducing seizures. Mechanistically, the KD may result in modifications in DNA methylation, regulation of glutathione levels, modulation of microRNAs targeting metabolic genes, and effects on epigenetic markers, potentially providing neuroprotective and anti-inflammatory effects.

Adipose tissue, obesity, appetite-regulating hormones, and inflammatory cytokines play crucial roles in KD-mediated metabolic dysfunction. The adipose tissue secretes leptin, which exerts anti-steatotic effects by reducing lipid accumulation and lipotoxicity. Adiponectin is another secretion of adipose tissue that protects coronary circulation, improves endothelial function, and decreases oxidative stress in the heart. Resistins play a vital role in the central nervous system. The KD also plays a key role in influencing the appetite-regulating hormones GDF15, ghrelin, and FGF21. The KD can reduce inflammation by inhibiting inflammasome activation, reducing pro-inflammatory cytokine levels, and facilitating anti-inflammatory effects under diverse pathological conditions. Moreover, the KD, characterized by elevated BHB and cytokine levels, exhibits anti-inflammatory effects, inhibits inflammasome activation, and relieves neuroinflammation. Therefore, hormones derived from adipose tissue or cytokines may be significant mediators of KD effects through mechanisms that remain poorly understood.

## Figures and Tables

**Table 1:** Diet composition for ketogenic therapy and rodent studies.

Diet of Humans and Rodents	Types of Diets	Carbohydrates (%)	Fats (%)	Proteins (%)	References/ Product No.
Standard American diet	regular diet	50–60	25–35	10–20	[14]
Human ketogenic diet therapy	classic long-chain triglyceride ketogenic diet	5–10	80–90	25–35	[10,15]
	medium-chain triglyceride ketogenic diet	10	70	20	[10,16]
	modified Atkins ketogenic diet	5–10	70–80	15–25	[10,17]
	low glycemic index treatment	10	60	30	[10,18]
Representative rodent diets for animal research (Research Diets Inc.)	normal chow diet (standard regular diet)	65–70	10–15	20	D12450
	high fat diet (HFD)	20–35	45–65	20	D12492, D12451
	ketogenic diet	0–5	80–90	10–20	D10070801, D05052004
	Control diet for ketogenic diet (high carbohydrate diet)	80	10	10	D10070802, D19082304

**Table 2:** Effect of ketogenic diet and associated metabolic changes on organ-specific diseases.

Organ-Specific Diseases	Effects of Ketogenic Diet	References
Non-alcoholic fatty liver disease	KD decrease fasting glucose and serum concentration, as well as levels of endogenous glucose production	[30]
	Decreases insulin levels and shows reduction in de-novo lipogenesis	[94]
	Rates of liver triglycerides hydrolysis upregulate due to increase production of hepatic ketones	[95]
	Increase in the FGF21mRNA level has also been observed in NAFLD	[96]
	IL-6 induces free fatty acid (FFA) release from visceral adipocytes, which in turn promotes diet-induced hepatic insulin resistance and steatosis	[97]
	KD increase the activity of forkhead box O3 (FOXO3) and p53, transcription factors	[98]
Cardiovascular diseases	KD dramatically increases myocardial uptake and utilization of glucose, lactate, and pyruvate	[37]
	Increased ketone body levels decreased the inhibitory effects of ketone bodies on fatty acid oxidation	[99]
	Circulating ketone bodies produced by the KD may improve myocardium functioning and can contribute to the treatment of cardiovascular system	[100]
	High adiponectin levels improved myocardial hypertrophy and diastolic dysfunction in HFpEF rats, regardless of their effect on blood pressure	[101]
	Adipose tissue derived adiponectins exert antioxidant effect on cardiomyocytes via downregulation of myocardial NADPH oxidase activity by preventing AMPK-mediated membrane	[102]
Sarcopenic obesity	KD could prevent loss of skeletal muscle during acute inflammation and preserve muscle mass	[103]
	KD enhance bioenergetic signaling to increase mitochondrial fat oxidation and endogenous antioxidant defenses while reducing inflammation	[104]
	KD reduced the expression of the gene encoding collagen, an extracellular matrix protein, in all muscles	[105]
	KD can lead to muscle atrophy involving factors such as hypercorticosteronemia, hyperinsulinemia, reduced insulin-like growth factor 1 (IGF-1), and oxidative stress	[106]
	Adipose tissue secreted leptin increases hepatic lipid oxidation and lipolysis in skeletal muscle	[107]
	In skeletal muscle, leptin alters lipid partitioning indicated by enhanced muscle fatty acid oxidation and reduced incorporation of fatty acids into triacylglycerols	[108]
Neuroinflammation	KD shows positive effects on neurological conditions, such as migraine, glaucoma, multiple sclerosis, Parkinson’s disease, and Alzheimer’s disease	[109]
	KD increase serum ketone levels which gradually improve seizure control	[110]
	Ketone bodies increased ATP generation, inhibit mitochondrial membrane transition pore and vesicular glutamate transporters, and helps in activating A1 receptors	[111]
	KD reduces intracellular reactive oxygen species and alleviates cellular metabolic stress, thereby enhancing fuel availability to neurons and decreasing neuronal excitability	[112]
	Leptin-deficient mice exhibit obesity, insulin resistance, and hyperphagia, showing the significant role of this adipose-derived hormone in controlling feeding behavior. Peripheral leptin affects the brain by binding to the choroid plexus, leading to its transport across the blood brain barrier	[113]
Cancer cachexia	Adipocytes secreted leptin upregulates the production of inflammatory cytokines. It acts on the hypothalamus to regulate the amount of energy stored in fat	[114]
	Circulating leptin levels are decreased in the setting of tumor-induced cachexia	[115]
	The activation of the ATP-dependent ubiquitin-proteasome proteolytic pathway (UPP) leads to the degradation of myofibrillar proteins and is essential in the process of muscle wasting	[116]
	Serum TNF-α is positively associated with serum FFA and accelerate WAT lipolysis in early stage of cachexia	[117]
	Early cachexia is identified by moderate reductions in adipose tissue mass, mild muscle wasting, and PKA-activated lipolysis without lipid accumulation in the liver and muscles	[118]

## Data Availability

No new data were created or analyzed in this study. Data sharing is not applicable to this article.
